# The long‐term survival of esophageal cancer in elderly patients: A multi‐center, retrospective study from China

**DOI:** 10.1002/cam4.5307

**Published:** 2022-10-10

**Authors:** Runhua Li, Yutong He, Xibin Sun, Ning Wang, Min Zhang, Kuangrong Wei, Huizhang Li, Peng Dong, Lingbin Du, Wanqing Chen

**Affiliations:** ^1^ Department of Cancer Prevention/Zhejiang Key Laboratory of Diagnosis and Treatment Technology on Thoracic Oncology (Lung and Esophagus) The Cancer Hospital of the University of Chinese Academy of Sciences (Zhejiang Cancer Hospital), Institute of Basic Medicine and Cancer (IBMC), Chinese Academy of Sciences Hangzhou Zhejiang P.R. China; ^2^ Cancer Institute, The Fourth Hospital of Hebei Medical University/The Tumor Hospital of Hebei Province Shijiazhuang Hebei China; ^3^ Henan Cancer Hospital Zhengzhou Henan China; ^4^ Peking University Cancer Hospital (Beijing Cancer Hospital) Beijing China; ^5^ Hubei Cancer Hospital Wuhan Hubei China; ^6^ Cancer Institute, Zhongshan City People's Hospital Zhongshan Guangdong China; ^7^ National Cancer Center/National Clinical Research Center for Cancer/Cancer Hospital, Chinese Academy of Medical Science and Peking Union Medical College Beijing P.R. China

**Keywords:** clinical risk factors, elderly patient, epidemiology, esophageal cancer, survival

## Abstract

**Background:**

Esophageal cancer (EC) often occurs in the elderly, and approximately 40% of patients are 70 years or older. To investigate the long‐term survival of EC in elderly patients, to provide a theoretical direction for better management and predicting survival of EC based on the hospital‐based multi‐center study in China.

**Methods:**

The study was conducted in 18 hospitals including 6 provincial hospitals, 8 municipal hospitals, and 4 county hospitals. We extracted information from medical record homepage, records of admission and discharge, and pathological diagnosis reports from the medical record department of the elderly patients at 70–84 years old to obtain the 3‐year and 5‐year overall survival (OS), and main associated factors, and to analyze the current therapeutic effect of different treatment options for elderly patients.

**Results:**

The 3‐year and 5‐year OS rate of the 1013 elderly patients was 44.8% and 32.8%, respectively. Their median survival was 28.00 months. The median survival of patients with squamous cell carcinoma was longer than that of other pathological type (squamous vs. other types: 31.00 vs. 20.00 months, *p* = 0.018). The median survival of patients with surgery only or combined therapy was longer than that of radiotherapy, chemotherapy, and no therapy (surgery only vs. combined therapy vs. radiotherapy vs. chemotherapy vs. no therapy: 56.00 vs. 33.00 vs. 26.00 vs.18.00 vs. 16.00 months, *p* < 0.001). The 5‐year OS rate of patients with highly differentiated cancer was higher than that of medium differentiated and poor differentiation/undifferentiated. In multivariate analysis, the older ages, pathological stage, were independent prognostic risk factors for poor EC survival. Treatment method was independent protective factors predictive of a good EC OS.

**Conclusions:**

The survival rate of the elderly EC patients was still low in China. Age, therapy method, and pathological stage were mainly associated with the survival rate of EC in elderly patients.

## Graphical abstract

We performed a multi‐center retrospective study to assess the survival of the elderly esophageal cancer patients. Our finding revealed that age, therapy method, and pathological stage were mainly associated with the survival of esophageal cancer in elderly patients.

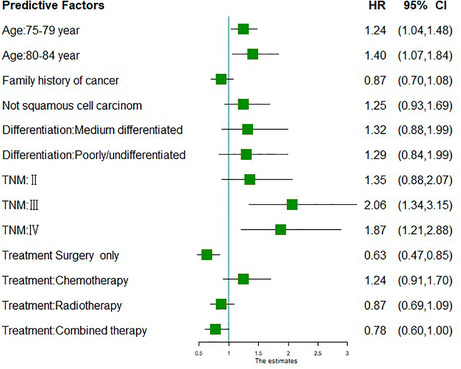

## 1 INTRODUCTION

Esophageal cancer (EC) is one of the high incidences of digestive tract malignant tumor, which ranks the sixth in the mortality rate in the world^1^, ^2^ and the fourth in China.^3^ Although the medical technology has made great progress and the study about EC is more and more accurate, the status quo in the treatment of EC is still unsatisfactory in developed and developing countries. In addition, the incidence trend of the disease is getting older and older, reaching the peak between 75 and 80 years old.^4^ It often occurs in the elderly, and approximately 40% of patients are 70 years or older.

Esophageal cancer is a worldwide health problem with a high mortality rate due to its natural history and the common diagnosis in advanced stages, especially on elderly patients.^5^ Therefore, it is vital to early detect EC in lower stage, which significantly improve survival. Esophageal squamous cell carcinoma is the most common histologic type worldwide including China. However, currently, adenocarcinoma subtype is dominant in the United States, Australia, the United Kingdom, and Western Europe (Finland, France, Norway), and squamous carcinoma has converted to the second place. Risk factors for squamous carcinoma mainly included gender and race, smoking, alcohol, genetics, obesity, gastroesophageal reflux disease and Barrett's Esophagus, diet, and nutrients such as chewers of areca nut and hot drinks.^6^, ^7^, ^8^, ^9^, ^10^, ^11^, ^12^

At present, there are great challenges in management and treatment of elderly patients with EC. A large amount of evidence recommended surgically based neoadjuvant chemoradiotherapy or radical concurrent chemoradiotherapy as the main treatment for advanced EC.^13^ For elderly patients, various functions are far inferior to those of young patients. Whether the above treatment methods are completely suitable for elderly patients still needs to be explored and discussed. The survival prognosis of patients after treatment is an important index for evaluating the therapeutic effect.

This study was based on elderly patients with EC were analyzed retrospectively, and studies the main related factors influencing the prognosis of survival in elderly patients, as well as the analysis of the current for elderly patients with treatment effects of different choice. We aimed to investigate influencing factors of the survival of EC in elderly patients, in order to provide a theoretical direction for better management and predicting survival of EC based on the hospital‐based multi‐center study in China.

## 2 METHODS

### 2.1 Data source

Data were collected from 18 hospitals of five regions including Beijing, provinces of Hebei, Henan, Zhejiang, Hubei, and Guangdong. These hospitals consisted of six provincial hospitals, eight municipal hospitals, and four county hospitals. All these hospitals are covered by population‐based cancer registries, and there are complete data of mortality and survival. Trained investigators excerpted information from homepage of medical record, records of admission and discharge, and pathological diagnosis reports from the department of medical records. Information included baseline characteristics: gender, age at diagnosis, marriage status, occupations, height and weight at admission, blood type, history of smoking, drinking, and family history of cancer (FHC); tumor‐related information: pathology, subsite, differentiation, and tumor node metastasis (TNM) stage of EC patients; treatment‐related information: surgery, radiotherapy, chemotherapy, chemoradiotherapy, combined therapy. For our study, we extracted the information of 1013 EC patients aged from 70 to 84 years old and diagnosed between 1 January 2011 and 31 December 2013 for survival analysis in this study.

### 2.2 Criteria of inclusion and exclusion

All included subjects met the following criteria: (1) all registered citizens diagnosed as EC; (2) patients diagnosed between 1 January 2011 and 31 December 2013; (3) patients who got first diagnosis and first treatment in these hospitals; (4) availability of complete clinical records.

Patients meeting the following criteria were excluded: (1) multiple primary or metastatic cancer; (2) nonlocal household records; (3) patients who underwent therapy (Surgery, radiotherapy, or chemotherapy) prior to admission to the above‐mentioned hospitals.

### 2.3 Exposure ascertainment and pathologic evaluation

According to the occupation information on the homepage of the medical records, occupations were divided into three categories, enterprise personnel, production and service personnel and farmers.

Body mass index (BMI) was calculated using the height and weight at admission, and according to Asian standard, BMI was categorized into three groups: underweight (<18.5 kg/m^2^), normal weight (18.5–23 kg/m^2^), and overweight or obese (≥23 kg/m^2^).

According to the admission records, patients were defined as smokers if smoking ≥100 cigarettes in one's lifetime. Drinkers were defined as individuals who drinking any alcoholic beverage one or more times a week. FHC was defined as any cancer among relatives with first‐degree relatives or second‐degree relatives. The first‐degree relatives included father, mother, and siblings; the second‐degree relatives were restricted in grandparents, uncle, and aunt. The pathological types and differentiation of all patients were confirmed by pathological examination. EC patients were staged according to the 2007 American Joint Committee on Cancer (AJCC) TNM classification. Classification of tumor sites and pathological types were according to ICDO‐3 coding.

### 2.4 Follow‐up

Patients were diagnosed between 1 January 2011 and 31 December 2013, and followed up until 31 December 2017. Both passive and active follow‐up methods to ascertain the vital status of patients were used in every hospital. The staff of hospital linked the patient records and death records of the population‐based cancer registries on the basis of identifiable information. Also, patients were followed up every 3 months by active methods in every hospital.

Survival time of patients who dead of EC was calculated from the time of diagnosis of EC until patients' death. Survival time of patients who were alive and censors was calculated from the time of diagnosis of EC until the last contact. Eventually, a total of 5283 patients with EC were included in the study. Up to 31 December 2017, there were 1333 survival cases, 3157 death cases, and 2575 cases of death due to EC. Seven hundred ninety‐three cases lost to follow‐up. Follow‐up rate was 85.0%. The study procedures have been described in detail elsewhere.^14^ We extracted the information of 1013 EC patients aged from 70 to 84 years old for survival analysis in this study.

### 2.5 Quality control

We standardized the format of the variables and applied automatic checking procedures to assess the data quality of each hospital.

#### 2.5.1 Checking of medical records

After investigation, we randomly selected 10% cases of all the 18 hospitals according to the table of random number methods. The medical records were checked by two other staff members, and the accuracy was all above 99.5%.

#### 2.5.2 Sampling of medical records

Medical records meeting the inclusion criteria were ranked by months, and these records were numbered according to the table of random number. Starting from any number and reading it in the same direction. The direction can be downward or upward, or left or right. In these numbers, the repeated number was excluded. Even‐numbered medical records were included in this cohort study, and odd numbers were excluded.

#### 2.5.3 Following up of these medical records

All hospitals followed the patients according to the above follow‐up methods and to minimize the loss of follow‐up and ensured the reliability of the data. The follow‐up rate remained above 85%.

### 2.6 Statistical analyses

The statistical analyses were performed using the IBM SPSS Statistics software (version 25.0; SPSS, Inc.). Qualitative variables were described by frequency and percentage. The forest map of associated factors with survival was drawn by the R software (version 4.1.1; The R Foundation for Statistical Computing). Rate of 3‐year and 5‐year overall survival (OS) and median survival time were analyzed by Kaplan–Meier method. The Cox's proportional hazard regression model was used to calculate hazard ratios (HR) and 95% confidence intervals (95% CI) of the univariate and multivariate survival analyses for mortality risk. Variables that were associated with the outcome variables at *p* less than 0.10 in the univariate logistic analysis model were included in the multivariate Cox's proportional hazard regression model. The statistical significance threshold was *p* < 0.05.

## 3 RESULTS

### 3.1 Clinical characteristics of elderly EC patients

There were 1013 EC patients aged from 70 to 84 years old, with a median age of 74 years old in our study. As of 31 December 2017, there were 345 (34.1%) patients who were still alive, 668 (65.9%) deaths. A total of 695 (68.6%) patients were male, and 318 patients (31.4%) were female. Patients who were married accounted for 95.5% (967/1013) in the elder EC patients. In elder patients, 513 were normal weight (18.5–23.0 kg/m^2^). There were 171 (16.9%), 192 (19.0%), 210 (20.7%), and 67 (6.6%) patients with A, B, O, and AB blood types, respectively. In total, 402 (39.7%) patients were smokers, and 308 (30.4%) were drinkers. Patients with a family history of any cancer accounted for 16.3% (165/1013) in elder EC patients.

Tumor characteristics include subsite, pathological types, differentiation, and TNM stage. As shown in Table 1, subsite was mainly in thoracic part (579, 57.2%) and the main pathological type was squamous cell carcinoma (826, 81.5%). 508 (50.1%) patients had stage III/IV at diagnosis. Moderately differentiated status was the most common histological type which accounted for 19.2% (194/1013) in the elder EC patients.

**TABLE 1:** Clinical characteristics of 1013 elderly patients with esophageal cancer.

Characteristics	Total	Deaths
	*N* (%)	*N* (%)
Total	1013 (100)	668 (100)
Gender		
Male	695 (68.6)	462 (69.2)
Female	318 (31.4)	206 (30.8)
Age (years)		
Median	74	
70–74	638 (63.0)	404 (60.5)
75–79	293 (28.9)	199 (29.8)
80–84	82 (8.1)	65 (9.7)
Marital status		
Married	967 (95.9)	639 (95.9)
Single and others	41 (4.1)	27 (4.1)
Occupation		
Enterprise personnel	86 (10.0)	53 (9.5)
Production and service personnel	246 (28.9)	159 (28.5)
Farmer and fisherman	527 (61.4)	346 (62.0)
BMI		
<18.5	95 (15.6)	62 (14.6)
18.5–23	353 (58.1)	252 (59.4)
≥23	160 (26.3)	110 (25.9)
Blood type		
A	171 (26.7)	107 (25.7)
B	192 (30.0)	134 (32.1)
O	210 (32.8)	129 (30.9)
AB	67 (10.5)	47 (11.3)
Smoking status		
Never smoker	598 (59.8)	392 (59.5)
Smoker	402 (40.2)	267 (40.5)
Drinking status		
Never drinker	693 (69.2)	451 (68.3)
Drinker	308 (30.8)	209 (31.7)
Family history of any cancer		
No	832 (83.4)	557 (84.8)
Yes	165 (16.5)	100 (15.2)
Subsite of tumor		
Cervical part	78 (8.3)	47 (7.6)
Thoracic section	579 (61.3)	369 (59.8)
Abdominal part	193 (20.4)	133 (21.6)
Overlapping lesion of esophagus	94 (10.0)	68 (11.0)
Pathological type		
Squamous cell carcinoma	826 (92.6)	533 (91.6)
Other types	66 (7.4)	49 (8.4)
Differentiation status		
Highly differentiated	57 (14.8)	29 (12.0)
Medium differentiated	194 (50.5)	127 (52.7)
Poorly and undifferentiated	133 (34.6)	85 (35.3)
TNM		
I	60 (11.8)	28 (8.7)
II	165 (32.5)	92 (28.7)
III	139 (27.4)	96 (29.9)
IV	144 (28.3)	105 (32.7)
Treatment		
No	165 (16.3)	118 (17.7)
Surgery	248 (24.5)	131 (19.6)
Chemotherapy	85 (8.4)	66 (9.8)
Radiotherapy	327 (32.3)	231 (34.6)
Combined therapy	186 (18.4)	122 (18.3)

Abbreviations: BMI, body mass index; TNM, tumor node metastasis.

### 3.2 OS rate and median survival time in elderly EC patients

The 3‐year and 5‐year OS rate of the 1013 elderly patients was 44.8% and 32.8%, respectively (Table 2). Their median survival was 28.00 months. The median survival time of patients with squamous cell carcinoma was longer than that of other pathological type (squamous vs. other types: 31.00 vs. 20.00, *p* = 0.018). The median survival time of patients with surgery only or combined therapy was longer than that of radiotherapy, chemotherapy, no therapy (surgery only vs. combined therapy vs. radiotherapy vs. chemotherapy vs. no therapy: 56.00 vs. 33.00 vs. 26.00 vs.18.00 vs. 16.00, *p* < 0.001). The 5‐year OS rate of patients with highly differentiated cancer was higher than that of medium differentiated and poor differentiation/undifferentiated (53.3% vs. 35.2% vs. 30.4%, *p* = 0.072). The 5‐year OS rate of patients with stage I cancer was higher than that of stage II and stage III and stage IV (53.4% vs. 42.5% vs. 29.2% vs. 26.5%, *p* = 0.072). The 5‐year OS rate of patients whose FHC was higher than that no history (31.1% vs. 41.8%, *p* = 0.05). There was no significant difference in the 5‐year OS rate between smokers and never smokers (smokers and never‐smokers: 32.6% vs. 33.3%, *p* = 0.609), and there was no significant difference in the 5‐year OS rate between drinkers and never drinkers (drinkers vs. never drinkers: 33.2% vs. 31.9%, *p* = 0.313).

**TABLE 2:** Overall survival rate and median survival time of 1013 elderly patients with esophageal cancer.

Characteristics	Overall survival rate (95% CI)		Median survival time (95% CI)	*p*
	3 year	5 year		
All patients	0.448	0.328	28 (24.24, 31.76)	
Age (year)				
70–74	0.487	0.356	35 (29.31, 40.69)	<0.001
75–79	0.389	0.306	22 (17.12, 26.89)	
80–84	0.300	0.196	20 (11.25, 28.75)	
Gender				
Male	0.443	0.325	27 (22.92, 31.08)	0.516
Female	0.458	0.334	32 (25.04, 38.96)	
Marital status				
Married	0.446	0.329	28 (10.35, 45.65)	0.526
Single and others	0.426	0.279	28 (24.22, 31.78)	
Occupation				
Enterprise personnel	0.453	0.341	33 (16.59, 49.41)	0.947
Production and service personnel	0.482	0.322	32 (22.77, 41.23)	
Farmer and fisherman	0.457	0.344	31 (25.75, 36.25)	
BMI (kg/m^2^)				
<18.5	0.368	0.312	21 (13.59, 28.41)	0.822
18.5–23.9	0.398	0.263	24 (21.20, 26.80)	
≥24	0.408	0.308	26 (19.87, 32.13)	
Blood type				
A	0.49	0.363	37 (24.57, 49.44)	0.155
B	0.431	0.294	28 (20.37, 35.64)	
O	0.481	0.375	33 (22.13, 43.88)	
AB	0.388	0.311	22 (14.99, 29.01)	
Smoking status				
Never smoker	0.453	0.326	31 (26.06, 35.94)	0.609
Smoker	0.442	0.333	26 (20.03, 31.97)	
Drinking status				
Never drinker	0.453	0.332	30 (25.43, 34.57)	0.313
Drinker	0.435	0.319	26 (19.50, 32.50)	
Family history of cancer				
No	0.436	0.311	27 (23.28, 30.72)	0.05
Yes	0.5	0.418	37 (25.39, 48.62)	
Subsite of tumor				
Cervical part	0.42	0.355	24 (16.65, 31.35)	0.115
Thoracic section	0.481	0.35	33 (27.34, 38.66)	
Abdominal part	0.411	0.299	25 (18.26, 31.75)	
Overlapping lesion of esophagus	0.371	0.258	23 (19.55, 26.45)	
TNM				
I	0.717	0.534	64 (56.03, 71.97)	<0.001
II	0.588	0.425	47 (36.48, 57.52)	
III	0.386	0.292	23 (19.40, 26.60)	
IV	0.352	0.265	22 (16.17, 27.83)	
Pathological type				
Squamous cell carcinoma	0.461	0.342	31 (26.50, 35.50)	0.018
Other types	0.354	0.243	20 (11.13, 28.87)	
Differentiation status				
Highly differentiated	0.614	0.533	64 (51.17, 76.83)	0.072
Medium differentiated	0.47	0.352	32 (24.03, 39.97)	
Poorly and undifferentiated	0.475	0.304	32 (17.97, 46.04)	
Treatment				
No	0.368	0.282	16 (10.05, 21.95)	<0.001
Surgery	0.602	0.469	56 (44.98, 67.02)	
Chemotherapy	0.257	0.206	18 (13.93, 22.07)	
Radiotherapy	0.401	0.271	26 (21.14, 30.86)	
Combined therapy	0.473	0.33	33 (24.37, 41.63)	

Abbreviations: BMI, body mass index; CI, confidence interval; TNM, tumor node metastasis.

### 3.3 Univariate and multivariate analysis of EC survival in elderly patients

The factors associated on univariate analysis with elderly EC survival are shown in Table 3. In the univariate analysis, advanced TNM stage (stage II: HR = 1.34, 95% CI = 0.88, 2.05; stage III: HR = 2.20, 95% CI = 1.44, 3.35; stage IV: HR = 2.38, 95% CI = 1.57, 3.61), nonsquamous cell carcinoma (HR = 1.41, 95% CI = 1.05, 1.89), medium (HR = 1.56, 95% CI = 1.04, 2.33) and poor differentiation (HR = 1.59, 95% CI = 1.04, 2.42), and elder patients (75–79 years: HR = 1.28, 95% CI = 1.08, 1.51; 80–84 years: HR = 1.61, 95% CI = 1.24, 2.10) were factors of poor prognosis, while FHC (HR = 0.81, 95% CI = 0.66, 1.00) and treatment methods (surgery only: HR = 0.51, 95% CI = 0.39, 0.65; combined therapy: HR = 0.72, 95% CI = 0.56, 0.93) were factors for good prognosis.

**TABLE 3:** Univariate and multivariate analysis between the characteristics and the survival in elderly patients with esophageal cancer.

Characteristics	Univariate			Multivariate		
	HR	95% CI	*p*	HR	95% CI	*p*
Gender						
Male	1	Reference	0.52			
Female	0.95	0.80, 1.12				
Age (years)						
70–74	1	Reference	<0.001	1	Reference	0.01
75–79	1.28	1.08, 1.51		1.24	1.04, 1.48	
80–84	1.61	1.24, 2.10		1.4	1.07, 1.84	
Marital status						
Married	1	Reference	0.531			
Single and others	1.13	0.77, 1.66				
Occupation						
Enterprise personnel	1	Reference	0.948			
Production and service personnel	1.01	0.74, 1.37				
Farmer and fisherman	0.98	0.73, 1.30				
BMI						
<18.5	1	Reference	0.826			
18.5–23	0.96	0.73, 1.27				
≥23	0.91	0.67, 1.25				
Blood type						
A	1	Reference	0.168			
B	1.2	0.93, 1.55				
O	0.99	0.77, 1.28				
AB	1.2	0.96, 1.51				
Smoking status						
Never smoker	1	Reference	0.78			
Smoker	1.04	0.89, 1.22				
Drinking status						
Never drinker	1	Reference	0.319			
Drinker	1.09	0.92, 1.28				
Family history of cancer						
No	1	Reference	0.05	1	Reference	0.358
Yes	0.81	0.66, 1.00		0.87	0.70, 1.08	
Subsite of tumor						
Cervical part	1	Reference	0.134			
Thoracic section	1.11	0.79, 1.54				
Abdominal part	1.21	0.83, 1.75				
Overlapping lesion of esophagus	1.18	0.80, 1.76				
Pathological type						
Squamous cell carcinoma	1	Reference	0.022	1	Reference	0.139
Other types	1.41	1.05, 1.89		1.25	0.93, 1.69	
Differentiation status						
Highly differentiated	1	Reference	0.025	1	Reference	
Medium differentiated	1.56	1.04, 2.33		1.32	0.88, 1.99	
Poorly and undifferentiated	1.59	1.04, 2.42		1.29	0.84, 1.99	
TNM						
I	1	Reference	<0.001	1	Reference	0.002
II	1.34	0.88, 2.05		1.35	0.88, 2.07	
III	2.2	1.44, 3.35		2.06	1.34, 3.15	
IV	2.38	1.57, 3.61		1.87	1.21, 2.88	
Treatment						
No	1	Reference	<0.001	1	Reference	0.001
Surgery only	0.51	0.39, 0.65		0.63	0.47, 0.85	
Chemotherapy	1.23	0.91, 1.66		1.24	0.91, 1.70	
Radiotherapy	0.87	0.69, 1.08		0.87	0.69, 1.09	
Combined therapy	0.72	0.56, 0.93		0.78	0.60, 1.00	

Abbreviations: BMI, body mass index; CI, confidence interval; HR, hazard ratio; TNM, tumor node metastasis.

**FIGURE 1:**
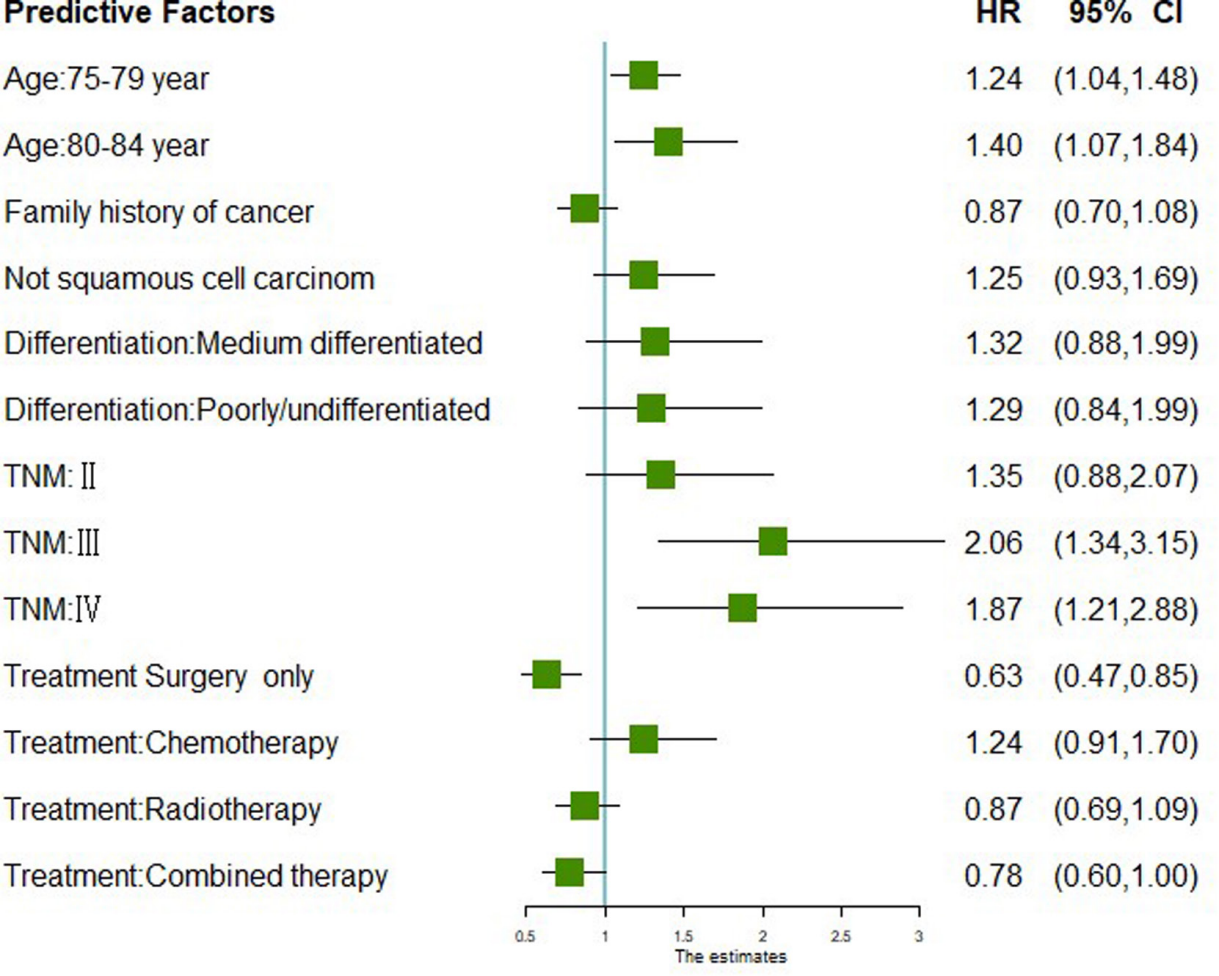
Forest plot of the significant risk factors related to the survival of esophageal cancer in elderly patients.

In multivariate analysis, the older ages (75–79 years: HR = 1.24, 95% CI = 1.04–1.48; 80–84 years: HR = 1.40, 95% CI = 1.07–1.84) and pathological stage (stage III: HR = 2.06, 95% CI = 1.34–3.15; stage IV: HR = 1.87, 95% CI = 1.21–2.88) were independent prognostic risk factors for poor EC survival. Treatment method (surgery only: HR = 0.63, 95% CI = 0.47–0.85; combined therapy: HR = 0.78, 95% CI = 0.60–1.00) was independent protective factors predictive of a good EC OS (Figure 1).

## 4 DISCUSSION

This study found that age, tumor stage, and treatment were independent risk factors affecting survival time of patients. The survival time of patients aged over 75 years is significantly shorter than that of patients aged under 75 years old. Elderly patients are often accompanied by organ dysfunction, decreased immunity, and various chronic diseases. When clinicians choose treatment strategies according to pathological characteristics and clinical stages of tumors and so on, the basic physiological status of patients gave enough attention and the significance of different treatment methods to the prognosis, survival, and quality of life of patients should also be considered. In the clinical treatment, it is common to choose relatively conservative treatment strategies when patients are too old, which may cause that some older patients do not receive more suitable treatment, thus affecting the survival time of patients. In this study, age is negatively correlated with survival time. Firstly, the patient's own physical condition is taken into account, and secondly, the choice of clinical treatment also affects the survival time of patients because they are too old.

The best treatment for elderly EC patients has always been controversial. Most scholars believe that surgery has great trauma to elderly patients and chemotherapy has many toxic and side effects, so radiotherapy is recommended for elderly patients with EC.^15^, ^16^ With the rapid development of minimally invasive surgical techniques and anesthesia techniques, the feasibility and safety of surgical treatment for elderly EC patients have been confirmed.^17^, ^18^ Lymph node dissection is an important step in EC surgery. Previous studies showed that the more lymph nodes dissected, the more accurate the postoperative staging was, and the better long‐term prognosis of patients had.^19^, ^20^ However, extensive lymph node dissection lengthens the operative time and increases intraoperative bleeding, resulting in increased perioperative complications and mortality.^21^ Elderly patients with EC have short life expectancy, poor postoperative recovery ability and high risk of perioperative death.^22^ Therefore, surgeons should consider the pros and cons of lymph node dissection in elderly patients.

Our study also suggests that surgery and combination therapy may have a significant survival benefit in elderly patients with EC. A large number of studies have basically confirmed that surgically based combination of radiotherapy and chemotherapy has significant efficacy for young patients with EC or patients with good physical fitness.^23^, ^24^ For advanced EC, some studies have advocated the use of radiotherapy alone or combination of radiotherapy and chemotherapy.^25^ A recent study in Japan showed that the 5‐year OS rate after esophageal resection for cancer in patients older than 70 years was similar to that in nonelderly patients,^26^ better than those of chemoradiotherapy alone.^27^ The surgery compared to nonsurgical treatment increased median OS among elder patients from 5 to 15 months in a European study.^18^ However, for elderly advanced EC patients, the poor physical quality, declined organ function, reduced tolerance of treatment and late‐onset characteristics of EC can affect the choice of treatment methods and treatment effect, which need more sample size and the research of higher quality to explore the suitable treatment methods in elderly EC patients.

The 5‐year survival rate of early EC is improved by more than 90% after the comprehensive therapy of surgery, radiotherapy, and chemotherapy, but the 5‐year survival rate of middle and advanced EC is not optimistic. EC in the elderly is mostly in the middle and late stage, with many complications, poor physique, and poor efficacy. Most patients seek medical treatment after obvious symptoms. After comprehensive treatment, the efficacy is also poor, and most patients die within 1 year. In this way, it is expected to strengthen the elderly tumor survey work in the future, early detection, early diagnosis, early reasonable treatment, in order to improve the survival period of the elderly EC.

In our study, it was found that the 5‐year survival rate of elderly patients with EC stage I was 53.4%, and the median survival time was 64 months while the 5‐year survival rate of EC stage IV was 26.5%, and the median survival time was 22 months. In our study, the 3‐year OS rate was 71.7% among Stage I patients, highly similar to 71.6% in metropolitan areas in Japan, which implies the importance for the detection and timely treatment for EC.^28^ TNM staging of EC is a general comprehensive judgment of the stage in the development of EC, which integrates three indicators (Tumor size, lymph node, metastasis) that have an important impact on the prognosis of EC patients, so it is an important factor affecting the treatment and prognosis of patients with EC.^5^, ^29^ The earlier the tumor stage is, the higher the surgical resection rate will be, the better the treatment effect will be. Therefore, early detection, early surgical resection, and comprehensive treatment are the main measures to improve the prognosis of esophageal carcinoma. The prognosis of EC is highly correlated with tumor stage, which suggests the importance of early detection of EC with lower stage and early diagnosis and treatment to prolong the survival time.

The occurrence of EC is related to environment, diet and lifestyle, poverty. Low nutrient, high‐calorie drinks, and food can increase the incidence of EC,^30^, ^31^, ^32^, ^33^, ^34^ and smoking and excessive drinking are also one of the important risk factors of EC.^35^, ^36^ The occurrence of EC is also related to the polymorphism of genetic loci, and thus, EC has genetic susceptibility.^37^ EC in China has obvious regional distribution characteristics of high incidence, mainly distributed in Taihang Mountain system, Qinling Mountain system, and Huaihe River system, followed by Guangdong and Fujian coastal areas.^38^, ^39^ The experience in the prevention and treatment of EC shows that the key strategy to EC prevention and treatment is early detection, early diagnosis, and early treatment. The combined operation techniques of endoscopic screening, iodine staining of esophageal mucosa and indicative biopsy are the most practical and effective methods at present.^40^ Wei et al.^41^ conducted a 10‐year follow‐up study on the study cohort of more than 45,000 people in Cixian County, Hebei province, a high‐incidence site of EC, and found that endoscopy as an early screening method for EC can effectively reduce the morbidity and mortality of esophageal squamous cell carcinoma. Since 2005, China's Ministry of Health has launched a population‐based endoscopic screening program in 11 high‐risk areas, which has been expanded to cover more than 110 cities and counties in 29 provinces in China. The implementation results of the program show that EC screening has a high detection rate, early diagnosis rate, and treatment rate, with good performance.^40^ Therefore, it is very necessary to strengthen the early detection, early diagnosis, and early reasonable treatment of EC, in order to improve the survival rate of EC in the elderly, especially on elderly population.

However, our study also had some limitations. Firstly, in the study, different levels of diagnostic evaluation and treatment in different hospitals may have influenced the results. In future studies, we will investigate more cases in city and county hospitals. Secondly, smoking, alcohol consumption status, and other variables associated with EC are self‐reported, which led to recall bias. As a result, people who have never smoked may be misclassified, especially those who have been ever smokers and stopped smoking for less than 15 years. In addition, the proportion of the missing data of some variables such as differentiation status was relatively high, but our previous study had verified that it was within a reasonable range.^14^ Further more detailed data are required to increase the precision of the effect for these exposures and explored more factors related to EC survival.

## 5 CONCLUSIONS

The survival rate of the elderly EC patients was still low. Age, therapy method, and pathological stage were mainly associated with the survival rate of EC in elderly patients. This study revealed important survival information of elderly EC patients in China and provided helpful information for their clinical management and early detection.

## AUTHOR CONTRIBUTIONS

**Runhua Li:** Conceptualization (equal); data curation (equal); formal analysis (lead); methodology (lead); visualization (lead); writing – original draft (lead); writing – review and editing (lead). **Yutong He:** Data curation (equal). **Xibin Sun:** Data curation (equal). **Ning Wang:** Data curation (equal). **Min Zhang:** Data curation (equal). **Kuang‐Rong Wei:** Data curation (equal). **Huizhang Li:** Data curation (equal). **Peng Dong:** Data curation (equal). **Lingbin Du:** Conceptualization (lead); data curation (equal); methodology (supporting); supervision (equal); writing – original draft (supporting); writing – review and editing (supporting). **Wanqing Chen:** Conceptualization (equal); data curation (equal); supervision (equal).

## FUNDING INFORMATION

This research was funded by Zhejiang Key Laboratory of Diagnosis and Treatment Technology on Thoracic Oncology (Lung and Esophagus).

## CONFLICT OF INTEREST

The authors declare no conflict of interest.

## ETHICS APPROVAL AND CONSENT TO PARTICIPATE

The study was conducted according to the guidelines of the Declaration of Helsinki and approved by the ethical review board of Zhejiang Cancer Hospital, the Fourth Hospital of Hebei Medical University, Henan Cancer Hospital, Beijing Cancer Hospital, Hubei Cancer Hospital, Zhongshan City People's Hospital, and National Cancer Center of Chinese Academy of Medical Science & Peking Union Medical College. All patients were given a general informed consent form upon admission to hospital, allowing their medical records and information to be used for scientific research in strict confidentiality and without physical harm in the study.

## CONSENT FOR PUBLICATION

Not applicable.

## Data Availability

The data supporting the conclusions of this article are included within the article. The datasets analyzed in this study are not publicly available due to regulations set out by Data Security Law of the People's Republic of China regarding data anonymization but are available from the corresponding author on reasonable request.
